# Quercetin: Mechanisms of Action, Clinical Evidence in Metabolic Syndrome, and Translational Opportunities in Food Preservation

**DOI:** 10.3390/molecules31162810

**Published:** 2026-08-12

**Authors:** Daniel A. Jacobo-Velázquez

**Affiliations:** Tecnologico de Monterrey, Escuela de Ingeniería y Ciencias, Campus Monterrey, Av. Eugenio Garza Sada 2501 Sur, Monterrey 64849, NL, Mexico; djacobov@tec.mx

**Keywords:** quercetin, plant metabolism, metabolic syndrome, bioavailability, nutraceutical, food preservation, active packaging

## Abstract

Quercetin is a plant-derived flavonol positioned at the interface of metabolic health and food preservation. This review integrates quercetin chemistry, plant biosynthesis and metabolism, production-relevant extraction and microbial synthesis, bioavailability, mechanisms of action, preclinical and clinical evidence in metabolic syndrome (MetS), and applications in clean-label food preservation. Experimental studies indicate that quercetin modulates obesity-associated inflammation, dyslipidemia, hepatic steatosis, insulin resistance, hypertension, endothelial dysfunction, and gut-barrier impairment through interconnected Nrf2/HO-1, NF-κB/NLRP3, AMPK/SIRT1, PI3K/Akt, eNOS/NO, lipid metabolism, and microbiota-related pathways. Human evidence is narrower and heterogeneous: modest reductions in systolic blood pressure constitute the most consistent signal, whereas effects on fasting glucose, lipids, inflammatory markers, endothelial function, liver fat, and body weight vary by population, formulation, dose, and duration. In food systems, quercetin has been investigated as an antioxidant, antimicrobial, antibiofilm agent, and photodynamic photosensitizer. It is incorporated into edible films, coatings, freshness indicators, and controlled-release packaging, although most evidence remains laboratory-scale. Key translational challenges include limited aqueous solubility, variable bioavailability, incomplete long-term safety evidence, matrix-dependent efficacy, sensory constraints, manufacturing scale-up, migration, and regulation. Overall, quercetin is promising, but clinical use and industrial deployment require formulation-specific, adequately powered human studies and validation in clinically relevant populations and under commercially realistic processing conditions.

## 1. Introduction

**Figure 1:**
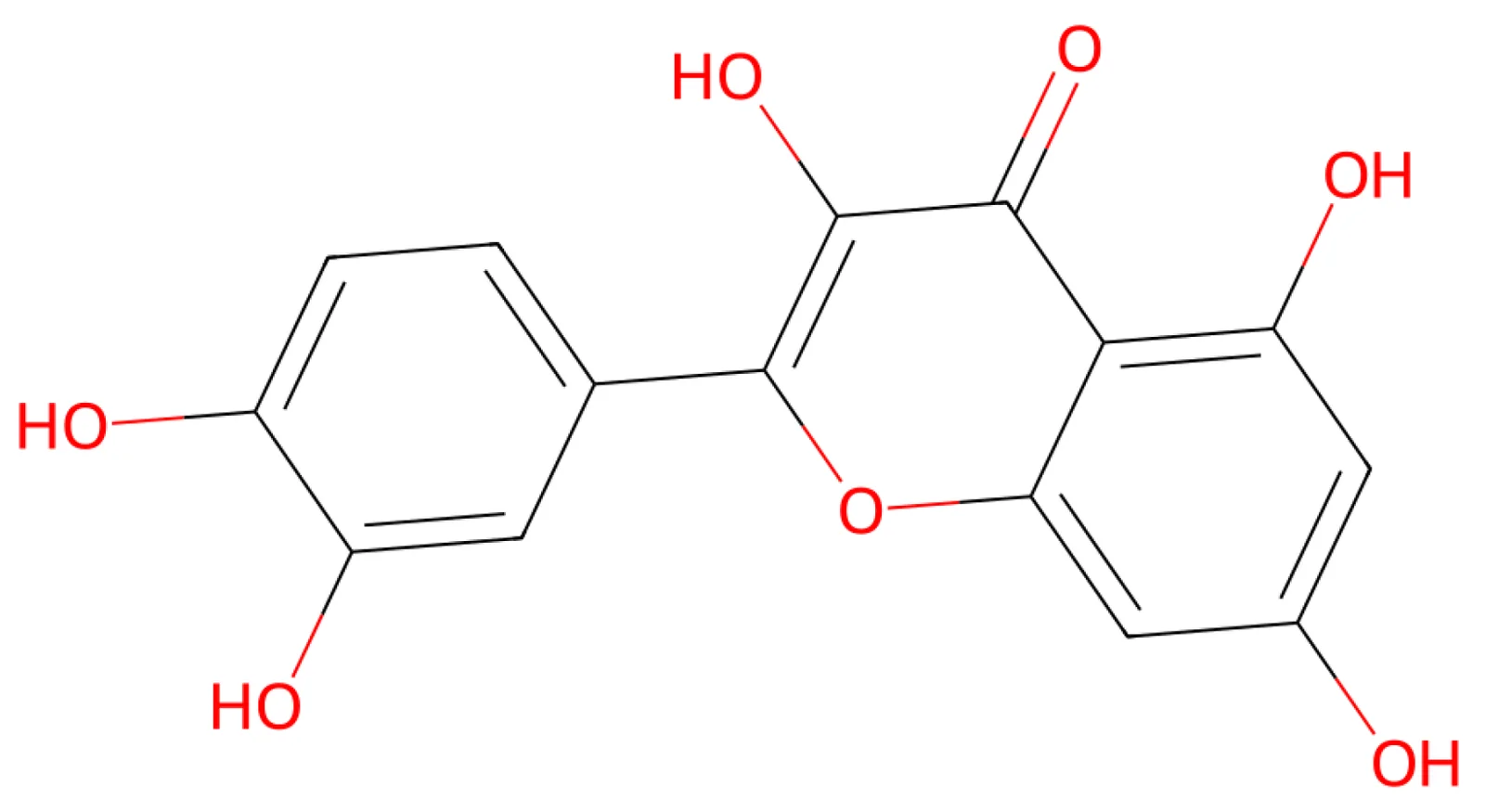
Chemical structure of quercetin (3,3′,4′,5,7-pentahydroxyflavone), a dietary flavonol with five hydroxyl groups and a conjugated C-ring that support radical-scavenging, metal-chelating, and redox-modulating activity.

Quercetin (3,3′,4′,5,7-pentahydroxyflavone; C_15_H_10_O_7_) is one of the most widely distributed dietary flavonols and is structurally characterized by a C_6_–C_3_–C_6_ flavonoid scaffold containing five hydroxyl groups and a 2,3-double bond conjugated with a 4-oxo function (Figure 1). These structural features confer metal-chelating capacity, hydrogen-atom and electron-donating activity, and resonance stabilization of radical intermediates, explaining much of its antioxidant behavior [1,2,3]. Quercetin occurs in foods primarily as glycosides, including quercetin glucosides, rutinoside (rutin), galactosides, and other conjugated derivatives. Onions, apples, berries, capers, leafy vegetables, tea, and many medicinal plants are common dietary sources, although the relative abundance of quercetin aglycone and glycosides varies markedly across cultivars, tissues, processing conditions, and extraction methods [4,5]. In contrast to hydroxycinnamic acids such as ferulic and chlorogenic acids, quercetin is a flavonol with a more hydrophobic, polyhydroxylated aromatic system; therefore, its biological and technological behavior depends strongly on glycosylation, methylation, conjugation, and interactions with food matrices.

Interest in quercetin has increased because it sits at the intersection of nutrition, pharmacology, food technology, and clean-label product development. From a health perspective, quercetin is investigated for anti-inflammatory, cardioprotective, antidiabetic, anti-obesity, hepatoprotective, neuroprotective, and antiviral activities [6,7,8]. Its translational relevance, however, is conditioned by limited aqueous solubility, extensive phase II metabolism, enterohepatic recycling, gut-microbial catabolism, and substantial differences among quercetin forms. Quercetin glucosides can be more efficiently absorbed than rutin or the aglycone in some settings, while interindividual differences in metabolism and microbiota can strongly influence exposure [9,10]. Modern formulation strategies such as cyclodextrin inclusion complexes, phytosomes, self-emulsifying systems, hybrid hydrogels, and colloidal delivery systems can markedly increase systemic exposure [11,12,13]. A 2025 systematic review of human intervention studies emphasized that the bioavailability of quercetin can increase dramatically with appropriate formulation, but also cautioned that improving solubility or stability alone does not guarantee improved bioavailability [13]. These considerations are central for interpreting clinical outcomes and for designing quercetin-enriched functional foods.

Metabolic syndrome (MetS) is a cluster of central obesity, insulin resistance or hyperglycemia, dyslipidemia, and elevated blood pressure that increases the risk of type 2 diabetes, cardiovascular disease, and metabolic dysfunction-associated steatotic liver disease (MASLD) [14,15]. For terminological consistency, MASLD is used throughout the narrative; nonalcoholic fatty liver disease (NAFLD) is retained only when reporting the terminology or diagnostic criteria used in the original publications. MetS is especially relevant for quercetin because the syndrome is driven by interacting oxidative, inflammatory, vascular, hepatic, adipose-tissue, and gut-microbiota pathways rather than by a single molecular defect. A multi-target phytochemical such as quercetin may therefore act as a metabolic modulator, not as a classical single-target drug. Previous reviews have typically examined quercetin pharmacology, glucolipid effects, bioavailability, extraction, or functional-food applications as separate topics [16,17]. Consequently, few have jointly appraised outcome-specific human evidence in MetS, the cross-talk among redox, inflammatory, metabolic, vascular, and gut-derived mechanisms, and the rapid development of food-preservation and active-packaging applications reported from 2020 to 2026. The present review addresses this gap through an integrated and critical synthesis that distinguishes preclinical plausibility from human efficacy and laboratory technological feasibility from commercial readiness. This dual perspective is relevant because quercetin may be delivered as an isolated nutraceutical, within a food matrix, or as a functional component of preservation and packaging systems.

This review is organized as follows. Section 2 describes the review design, literature search, eligibility criteria, record management, and narrative-synthesis approach. Section 3 evaluates preclinical and clinical evidence for quercetin in MetS and related disorders. Section 4 integrates the principal molecular mechanisms of action. Section 5 critically compares quercetin-based food-preservation strategies, while Section 6 examines plant biosynthesis, metabolism, extraction, and microbial production. Section 7 summarizes the principal conclusions, translational limitations, and future research priorities.

## 2. Materials and Methods

### 2.1. Review Design and Scope

This study was designed as a narrative review integrating three complementary areas of quercetin research: (i) preclinical and clinical evidence related to metabolic syndrome (MetS) and its associated disorders; (ii) applications of quercetin in food preservation, including antimicrobial, antibiofilm, antioxidant, photodynamic, coating, and active-packaging strategies; and (iii) plant metabolism, biosynthesis, extraction, and production of quercetin. The review was intended to provide a critical and translational synthesis rather than an exhaustive systematic review or quantitative meta-analysis. Recent evidence was prioritized, while earlier seminal studies were retained when required to establish historical, clinical, or mechanistic context.

### 2.2. Literature Search

The literature base assembled during preparation of the original manuscript was updated and standardized during revision to improve reproducibility and ensure that recent evidence was considered. PubMed and Scopus were searched on 6 August 2026 for records published from 1 January 2020 through that date. The record counts and selection process reported below correspond to this updated search. The search strategy comprised three thematic blocks and required “quercetin” to appear in the article title. In PubMed, thematic terms were searched in the Title/Abstract fields, whereas in Scopus they were searched in the Title, Abstract, and Keywords fields.

The metabolic-health block combined “quercetin” with “metabolic syndrome,” obesity, adiposity, dyslipidemia, hyperglycemia, diabetes, “insulin resistance,” hypertension, “blood pressure,” NAFLD, MASLD, “hepatic steatosis,” endothelial, or microbiota. The food-preservation block combined “quercetin” with “food preservation,” antimicrobial, antibiofilm, photodynamic, “active packaging,” “intelligent packaging,” “edible film,” coating, “shelf life,” or “lipid oxidation.” The plant and production block combined “quercetin” with “plant metabolism,” “plant biosynthesis,” “flavonol synthase,” glycosylation, “onion skin,” extraction, “subcritical water,” “process intensification,” “microbial synthesis,” “metabolic engineering,” or fermentation.

PubMed searches were limited to English-language records indexed as Journal Article, Review, or Meta-Analysis. Preprints, editorials, letters, comments, published errata, retracted publications, and retraction notices were excluded. Scopus records were restricted after export to those classified as Article or Review. Articles in press were retained when they were classified as Article or Review and had an assigned publication year within the predefined date range. No language filter was applied at the Scopus search stage; however, only English-language publications were eligible for inclusion.

### 2.3. Eligibility Criteria and Evidence Prioritization

Peer-reviewed publications were considered eligible when they contributed directly to at least one of the three thematic areas covered by the review. For clinical evidence, randomized or controlled human intervention studies and systematic reviews, meta-analyses, or umbrella reviews evaluating clinically relevant cardiometabolic outcomes were prioritized. Relevant outcomes included body weight and adiposity, glucose homeostasis and insulin resistance, blood pressure, lipid profiles, inflammatory biomarkers, endothelial function, hepatic steatosis, and liver-function markers.

For mechanistic evidence, in vivo and cellular studies were considered when they directly examined MetS-related pathways or outcomes, including oxidative stress, inflammation, glucose and lipid metabolism, endothelial function, mitochondrial regulation, hepatic lipid accumulation, adipose-tissue remodeling, or gut-microbiota interactions. Food-preservation studies were retained when they evaluated antimicrobial, antibiofilm, antioxidant, photodynamic, active-packaging, edible-film, coating, controlled-release, freshness-monitoring, or shelf-life outcomes in food-relevant microorganisms, matrices, or packaging systems. Plant and production studies were considered when they directly addressed quercetin biosynthesis, plant metabolism, glycosylation, extraction, recovery from plant by-products, fermentation, microbial production, or process intensification.

Records were excluded when they were not peer reviewed, were conference abstracts without a complete report, represented editorials, letters, corrections, retractions, or other ineligible document types, were outside the predefined thematic scope, or did not provide directly interpretable mechanistic, clinical, technological, or production-related information. When multiple publications addressed a similar question, priority was given to studies with greater methodological relevance, direct assessment of the outcomes of interest, more recent evidence, and limited redundancy with publications already selected. Earlier publications were retained when they represented seminal clinical studies, established foundational mechanisms, or provided essential historical context.

### 2.4. Record Management and Literature Selection

PubMed citation metadata were retrieved using PMID, DOI, and title information, whereas Scopus records were exported in CSV format using EID, DOI, and title information. Record management and deduplication were performed using Python 3.12.13. Records were first deduplicated within each database using PMID or EID and subsequently using normalized DOI or exact normalized title. Remaining duplicates between PubMed and Scopus were identified using normalized DOI or exact normalized title. DOI normalization included conversion to lowercase and removal of URL or “doi:” prefixes and trailing punctuation. Title normalization included Unicode normalization, conversion to lowercase, removal of punctuation, and consolidation of whitespace.

Across the three thematic blocks, 1107 records were retrieved from PubMed and 2096 from Scopus, resulting in 3203 database records. Twenty-eight Scopus records that were not classified as Article or Review were removed before deduplication, leaving 3175 records. Deduplication removed 40 records within PubMed, 126 within Scopus, and 1036 overlapping records between the databases. Thus, 1202 duplicate records were removed, leaving a candidate literature pool of 1973 unique records.

**Figure 2:**
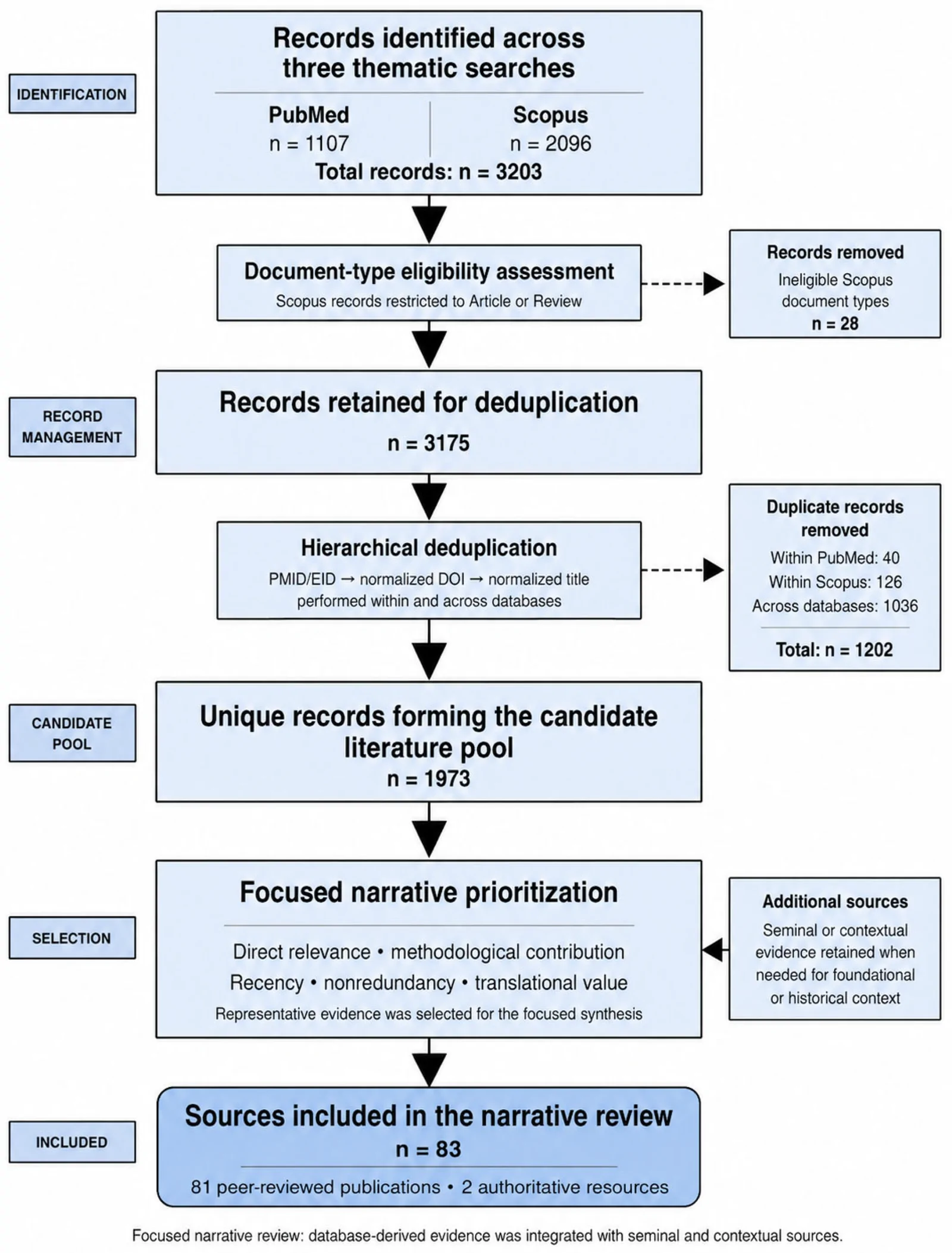
Flow diagram of literature identification, record management, and selection for the focused narrative review. PubMed and Scopus searches identified 3203 records. After removal of 28 records with ineligible document types and 1202 duplicates, 1973 unique records formed the candidate literature pool. Evidence was prioritized according to direct relevance, methodological contribution, recency, nonredundancy, and translational value. Database-derived evidence and additional seminal or contextual sources were integrated, resulting in 83 sources included in the final narrative synthesis.

Because this was a focused narrative review, the 1973 unique records constituted a candidate literature pool rather than a set intended for exhaustive systematic screening. Records were prioritized according to their direct relevance to the three thematic domains, methodological contribution, recency, nonredundancy, complementarity with the evidence already selected, and translational value. Additional seminal or contextual sources were retained when required to support chemical characterization, historical evidence, bioavailability, foundational clinical findings, or other essential background information. After integration of the prioritized database-derived evidence and additional contextual sources, the final manuscript included 83 sources, comprising 81 peer-reviewed publications and two authoritative reference resources. The literature-identification and selection process is summarized in Figure 2.

### 2.5. Data Extraction and Narrative Synthesis

For studies summarized in the tables and main text, relevant information was extracted according to the evidence domain. For in vivo studies, the extracted information included the experimental model, approximate sample size, intervention characteristics, metabolic condition, principal outcomes, and proposed mechanisms. For clinical studies and quantitative evidence syntheses, the population, study design, quercetin source or formulation, dose, intervention duration, sample size, cardiometabolic outcomes, and principal findings were considered. For food-preservation studies, the food matrix or experimental system, quercetin form or delivery strategy, target microorganism or preservation mechanism, and principal technological outcome were recorded.

Evidence was synthesized narratively and organized by thematic domain. Human randomized trials and quantitative evidence syntheses were given greater weight when evaluating clinical efficacy, whereas animal and cellular studies were used primarily to support biological plausibility and mechanistic interpretation. Food-preservation evidence was compared according to the maturity, advantages, limitations, and potential practical application of each strategy. Because of the breadth and heterogeneity of the included evidence, no quantitative meta-analysis or formal risk-of-bias assessment was performed.

### 2.6. Use of Generative Artificial Intelligence

Generative artificial intelligence (ChatGPT 5.5, OpenAI) was used as a support tool during manuscript preparation to assist with initial drafting, organization of content, language refinement, structuring of database search strategies, and computational record management and deduplication. The author reviewed and validated the search parameters, eligibility criteria, source selection, scientific interpretation, cited references, figures, tables, and final manuscript. All final decisions regarding the inclusion and interpretation of the evidence were made by the author, who assumes full responsibility for the accuracy and integrity of the manuscript.

## 3. Quercetin as a Nutraceutical for Metabolic Syndrome

Quercetin has been evaluated in experimental models and human studies for individual components of metabolic syndrome, including obesity, dyslipidemia, hyperglycemia, hypertension, and hepatic steatosis. Preclinical evidence is extensive and mechanistically coherent, whereas clinical evidence is promising but more variable, reflecting differences in dose, formulation, baseline risk, duration, population characteristics, and endpoints. Table 1 and Table 2 summarize representative in vivo studies, clinical trials, and meta-analyses, with emphasis on recent evidence from 2020 to 2026.

### 3.1. Anti-Obesity and Anti-Adiposity Effects

#### 3.1.1. Evidence from Animal Studies

In animal models of diet-induced obesity and metabolic syndrome, quercetin consistently reduces weight gain, visceral adiposity, adipocyte hypertrophy, macrophage infiltration, and inflammatory signaling. Earlier rodent studies established that quercetin attenuates several MetS features in high-carbohydrate/high-fat or genetic obesity models, including adiposity, blood pressure, triglycerides, hepatic inflammation, and oxidative stress [18,19]. Subsequent mechanistic work connected these effects to AMPKα1/SIRT1 activation, reduced adipose-tissue macrophage infiltration, and modulation of gut microbiota and gut–liver axis signaling [21,22]. Recent preclinical studies add that quercetin can improve HFD-induced obesity through gut microbiota remodeling, enhanced barrier function, and favorable metabolite shifts [29]. Moreover, quercetin appears to influence energy expenditure by activating brown adipose tissue thermogenesis and promoting white-adipose-tissue browning in HFD-induced obese mice [30].

#### 3.1.2. Evidence from Human Studies

Clinical evidence for weight loss is less robust than animal evidence. A pooled analysis of RCTs did not show a strong overall anti-obesity effect, although subgroup analyses suggested modest body-weight reductions when interventions used higher doses, longer duration, or crossover designs [49]. In elderly patients with MetS, however, quercetin supplementation for three months reduced body weight and BMI alongside blood pressure, LDL-C, fasting insulin, and 2 h glucose [40]. In the 2024 crossover trial of participants diagnosed with NAFLD under the original study criteria, 500 mg/day quercetin for 12 weeks produced modest reductions in body weight and BMI and lowered intrahepatic lipid content [46]. Thus, quercetin should not be framed primarily as a weight-loss agent; rather, its anti-obesity relevance lies in improving adipose inflammation, liver fat, insulin sensitivity, and vascular risk in metabolically vulnerable populations.

### 3.2. Anti-Dyslipidemic, Hepatoprotective, and Cardioprotective Effects

#### 3.2.1. Evidence from Animal Studies

Quercetin improves lipid handling in the liver, circulation, and arterial wall. In Western-style and high-fat diet models, quercetin attenuated hepatic triglyceride accumulation, reduced steatosis, and regulated genes involved in lipid transport and oxidation [20,23]. In ApoE−/− mice, quercetin reduced atherosclerotic plaque burden and inflammatory signaling through inhibition of Galectin-3/NLRP3 pathways, supporting a cardioprotective role beyond simple lipid lowering [28]. The protective effects observed in diet-induced hepatic-steatosis models appear to involve both hepatic mechanisms, including VLDL assembly and lipophagy, and extrahepatic mechanisms, including gut-microbiota modulation and attenuation of metabolic endotoxemia [22,23,29]. These findings are relevant to MASLD as a hepatic manifestation of metabolic dysfunction.

#### 3.2.2. Evidence from Human Studies

Human trials and meta-analyses indicate that quercetin may improve selected lipid and liver-related endpoints, but effects vary across populations. The 2020 meta-analysis by Tabrizi et al. found reductions in total cholesterol, LDL-C, and CRP, while effects on triglycerides, HDL-C, IL-6, and TNF-α were less consistent [41]. The 2024 dose–response meta-analysis of 20 RCTs reported no pooled effect on triglycerides or HDL-C but found reductions in fasting glucose and systolic blood pressure [45]. The most directly relevant liver trial is the 2024 randomized crossover study of participants diagnosed with NAFLD under the original study criteria, in which quercetin reduced MRI-PDFF-measured intrahepatic lipid content from 11.5% to 9.6%, while placebo caused almost no change [46]. A 2025 MASLD meta-analysis integrating seven RCTs reported improvements in liver enzymes, CRP, total cholesterol, LDL-C, triglycerides, and HDL-C, although the authors emphasized that evidence certainty remains preliminary [47]. These results support a cautious but positive interpretation: quercetin is more likely to improve cardiometabolic risk clusters than isolated lipid endpoints in all populations.

### 3.3. Anti-Diabetic and Glycemic Control Effects

#### 3.3.1. Evidence from Animal Studies

In diabetic and insulin-resistant animals, quercetin improves glucose homeostasis through multiple complementary mechanisms. In fructose/STZ-induced diabetic rats, quercetin lowered fasting glucose and HbA1c, restored pancreatic antioxidant defenses, and modulated enzymes involved in carbohydrate metabolism [24]. In obese diabetic rats, quercetin reduced glucose and lipid abnormalities and protected liver and kidney tissues, effects associated with AMPK/autophagy signaling [27]. Mechanistically, these outcomes are consistent with enhanced PI3K/Akt signaling, increased GLUT4 translocation, reduced gluconeogenesis, improved mitochondrial function, and suppression of oxidative-inflammatory damage in insulin-sensitive tissues.

#### 3.3.2. Evidence from Human Studies

Clinical findings on glycemic control are mixed. A meta-analysis of RCTs found no strong overall improvement in fasting glucose, HOMA-IR, or HbA1c, but subgroup analyses suggested that interventions lasting at least eight weeks or using doses of at least 500 mg/day may reduce fasting glucose and insulin [48]. The 2024 dose–response meta-analysis found a significant reduction in fasting blood glucose among adults receiving quercetin, but not consistent changes in all MetS components [45]. In PCOS, a condition characterized by insulin resistance and cardiometabolic risk, quercetin improved adiponectin-mediated insulin sensitivity and metabolic/hormonal parameters in randomized trials [37,38]. These results suggest that quercetin’s glycemic effects may depend on baseline insulin resistance, duration, and bioavailability rather than dose alone.

### 3.4. Antihypertensive and Vascular Effects

#### 3.4.1. Evidence from Animal Studies

Quercetin has one of its clearest mechanistic profiles in vascular biology. In spontaneously hypertensive rats, both quercetin and quercetin-derived metabolites lowered blood pressure and improved vascular function [25,26]. Lin et al. showed that quercetin improved endothelial function through promotion of autophagy, while the metabolite study indicated that physiologically relevant circulating metabolites can contribute to antihypertensive effects [25,26]. In L-NAME-induced hypertension, acute quercetin administration reduced blood pressure, supporting direct vascular or NO-related actions [31]. These findings collectively link quercetin to improved endothelial nitric oxide availability, lower oxidative stress, decreased vascular inflammation, and improved vasorelaxation.

#### 3.4.2. Evidence from Human Studies

Clinical support for blood-pressure effects is stronger than for several other MetS endpoints. Early RCTs found that quercetin reduced blood pressure in hypertensive or higher-risk adults [32,33,35]. Bioavailability-optimized isoquercitrin also improved endothelial function in volunteers at risk of cardiovascular disease, highlighting the importance of formulation [39]. Meta-analyses published after 2020 reinforce this pattern: quercetin significantly reduces systolic blood pressure, while diastolic effects are smaller or subgroup-dependent [42,44,45]. This supports positioning quercetin as a vascular-support nutraceutical in MetS, particularly when combined with delivery systems that improve systemic exposure.

**Figure 3:**
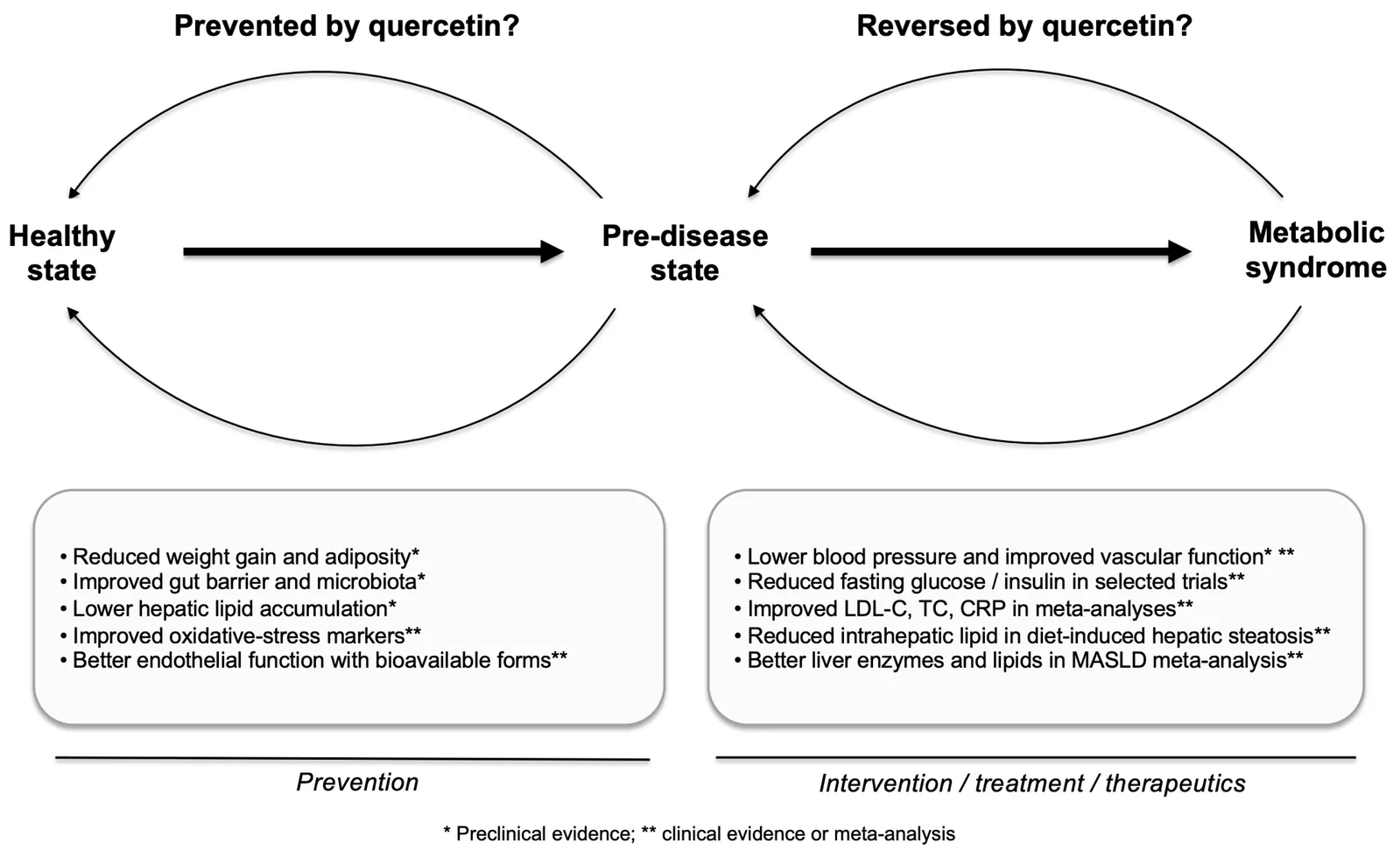
Proposed model for preventive and therapeutic effects of quercetin or quercetin-rich foods/supplements against metabolic syndrome based on in vivo studies (*) and clinical studies or meta-analyses (**). Abbreviations: CRP = C-reactive protein; LDL-C = low-density lipoprotein cholesterol; MASLD = metabolic dysfunction-associated steatotic liver disease; TC = total cholesterol. This preventive/therapeutic model has previously been applied to dietary phenolics such as chlorogenic acid [50], ferulic acid [51], and to food juices [52,53].

Figure 3 summarizes how quercetin may act at two stages of metabolic deterioration. In a pre-disease state, quercetin may help preserve metabolic resilience by reducing adiposity, oxidative stress, gut-barrier dysfunction, and early hepatic lipid accumulation. In established MetS or related disorders, quercetin may act as an adjunct intervention by improving blood pressure, fasting glucose, lipids, hepatic fat, inflammatory biomarkers, and endothelial function. The clinical evidence is not uniform, but recent RCTs and meta-analyses indicate that the most consistent translational signals involve systolic blood pressure, fasting glucose, selected lipid markers, and liver fat or liver-function endpoints.

### 3.5. Critical Appraisal of Clinical Evidence, Safety, and Translational Relevance

Within this narrative appraisal, which is not a formal GRADE assessment, the most consistent human signal is a modest reduction in systolic blood pressure, supported by several trials and meta-analyses [32,33,34,35,40,42,44,45]. Evidence for fasting glucose is suggestive but heterogeneous [40,45,48], as are the signals for total and LDL cholesterol and CRP [41,47] and for hepatic fat and liver enzymes [46,47]. Evidence remains inconsistent or insufficient for body weight and waist circumference, triglycerides, HDL-C, HbA1c or HOMA-IR, and diastolic blood pressure [44,45,48,49]. Therefore, no metabolic outcome can yet be regarded as conclusively established across populations and formulations.

The clinical studies varied in quercetin source and form, including pure quercetin, quercetin-rich onion-skin extract, and isoquercitrin; daily dose, ranging from approximately 150 mg to 1 g in the trials summarized here; duration, generally from 28 days to 3 months; trial design; baseline cardiometabolic phenotype; and endpoint definition [32,33,34,35,36,37,38,39,40,46]. Many trials were small and short. These differences, together with overlap among quantitative reviews and subgroup-dependent findings, limit precision and generalizability. Current evidence therefore supports, at most, a formulation- and phenotype-dependent adjunctive role; it does not justify treating quercetin as a stand-alone therapy or assuming equivalent efficacy among preparations.

Long-term safety remains a major unresolved issue. Adverse-event reporting was not a primary focus in most efficacy studies summarized here. The 12-week trial using NAFLD terminology and eligibility criteria reported no intervention-related adverse events [46], but this finding cannot establish the safety of chronic high-dose or bioavailability-enhanced supplementation. Future trials should prospectively record adverse events, adherence, renal and hepatic clinical chemistry, concomitant medication use, and potential supplement–drug interactions, while linking outcomes to plasma metabolites and formulation-specific exposure. Other priorities include adequately powered, preregistered, longer-duration trials, standardized MetS outcomes, direct comparison of formulations, and stratification by baseline cardiometabolic phenotype.

## 4. Mechanisms of Action of Quercetin in Metabolic Syndrome

**Figure 4:**
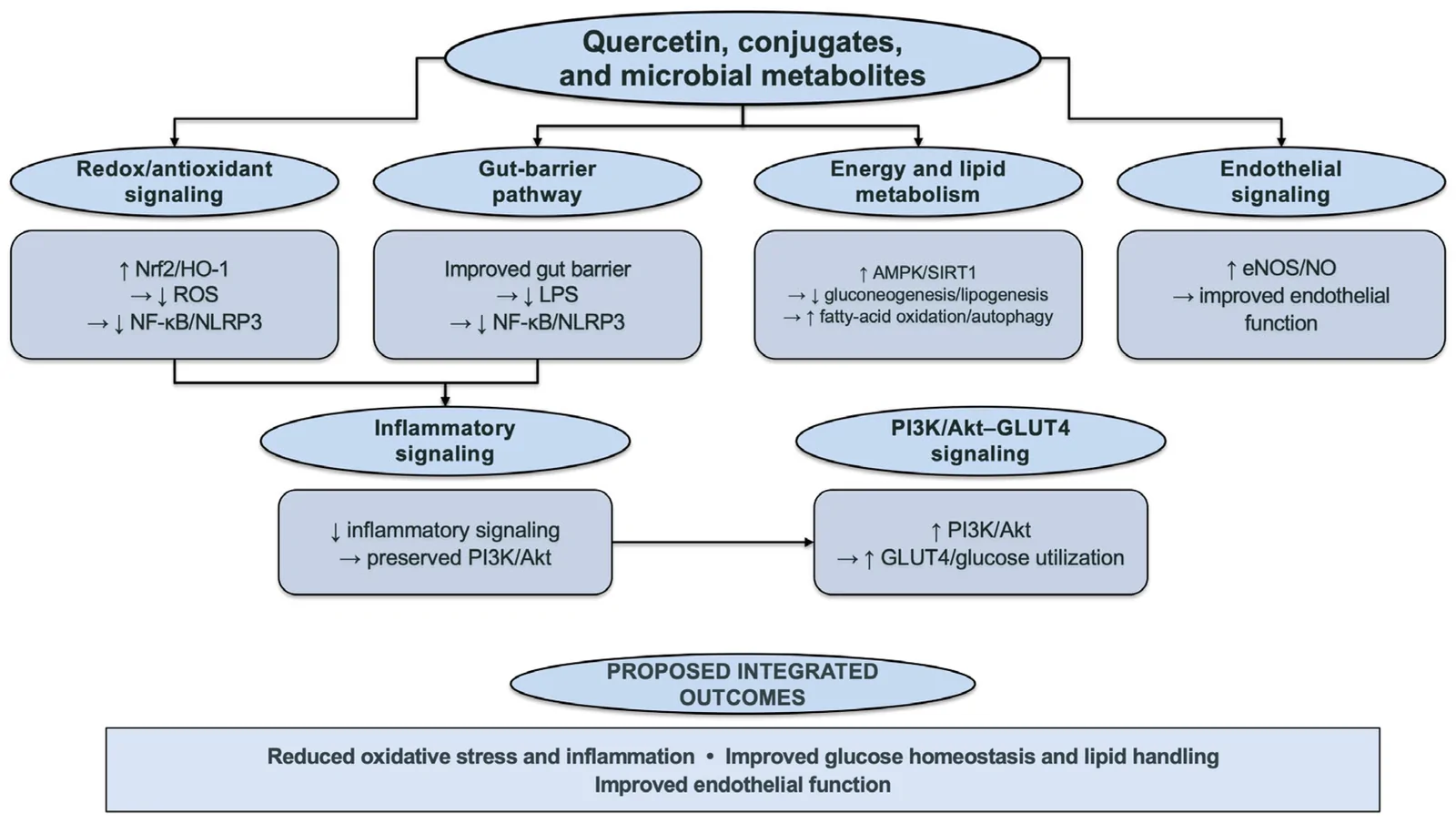
Proposed integrated mechanistic framework for quercetin in metabolic syndrome. Quercetin, circulating conjugates, and microbial catabolites may couple Nrf2/HO-1-mediated redox control, NF-κB/NLRP3 inflammatory signaling, AMPK/SIRT1 and PI3K/Akt metabolic regulation, eNOS/NO-dependent vascular function, and gut-barrier/microbiota effects. Abbreviations: AMPK = AMP-activated protein kinase; eNOS = endothelial nitric oxide synthase; GLUT4 = glucose transporter type 4; HO-1 = heme oxygenase 1; LPS = lipopolysaccharide; NF-κB = nuclear factor kappa B; NLRP3 = NOD-like receptor family pyrin domain-containing protein 3; NO = nitric oxide; Nrf2 = nuclear factor erythroid 2-related factor 2; PI3K/Akt = phosphoinositide 3-kinase/protein kinase B; ROS = reactive oxygen species; SIRT1 = sirtuin 1.

Quercetin’s bioactivity in MetS arises from an interlocking network of mechanisms rather than from a single pharmacological target. Its catechol B-ring, 3-hydroxyl group, and conjugated C-ring support redox activity, while its metabolites interact with inflammatory, vascular, metabolic, and microbiota-related pathways [17,54,55]. Figure 4 summarizes the major mechanisms proposed to explain the effects observed in animal and human studies.

### 4.1. Antioxidant Activity

Oxidative stress is a driver of insulin resistance, endothelial dysfunction, adipose inflammation, and hepatic steatosis. Quercetin can directly scavenge reactive oxygen and nitrogen species, chelate transition metals, inhibit lipid peroxidation, and modulate endogenous antioxidant defenses [3]. In cell and animal models, quercetin activates nuclear factor erythroid 2-related factor 2/antioxidant response element (Nrf2/ARE) signaling, increasing the expression of heme oxygenase-1 (HO-1), NAD(P)H quinone oxidoreductase 1 (NQO1), glutathione-related enzymes, superoxide dismutase, and catalase. In clinical trials, oxidative-stress endpoints are less consistent; nevertheless, meta-analytic evidence indicates improvements in selected antioxidant-capacity markers, especially ferric reducing antioxidant power (FRAP), whereas effects on malondialdehyde (MDA) and total antioxidant capacity (TAC) are more variable [43]. This discrepancy likely reflects differences between direct antioxidant chemistry in vitro and the metabolite-mediated, low-concentration signaling effects that occur in humans. Overall, evidence for direct antioxidant chemistry and Nrf2 activation is strong in experimental systems, whereas human biomarker responses remain inconsistent; future studies should relate standardized redox biomarkers to circulating quercetin metabolites and clinically relevant outcomes.

### 4.2. Anti-Inflammatory Effects

Low-grade inflammation links adipose expansion, insulin resistance, endothelial injury, and MASLD. Quercetin inhibits NF-κB and MAPK activation, reduces proinflammatory cytokines such as TNF-α and IL-6, and suppresses NLRP3 inflammasome signaling [16,28]. In ApoE−/− mice, inhibition of Galectin-3/NLRP3 signaling was associated with reduced atherosclerotic inflammation [28]. In obesity and hepatic-steatosis models, quercetin reduces adipose-tissue macrophage infiltration and gut-derived inflammatory activation [21,22,29]. Clinically, reductions in CRP appear more consistent than reductions in individual cytokines, which may reflect both lower assay sensitivity and heterogeneity of inflammatory status at baseline [41,47]. Overall, inhibition of NF-κB/NLRP3-linked inflammation is consistent across experimental models, but human evidence is largely limited to variable systemic biomarkers and does not yet establish pathway-specific effects; trials stratified by baseline inflammatory status are needed.

### 4.3. Regulation of Glucose Metabolism

Quercetin regulates glucose metabolism through hepatic and peripheral mechanisms. In diabetic rodents, quercetin lowers gluconeogenic output, improves insulin signaling, supports GLUT4 translocation, and reduces oxidative damage in pancreatic and insulin-sensitive tissues [24,27]. Mechanistically, activation of AMPK and PI3K/Akt pathways can increase glucose uptake and glycogen synthesis while suppressing FoxO1-dependent gluconeogenic genes. In humans, these effects are more likely to emerge in insulin-resistant populations or with sufficient dose/duration; this interpretation is consistent with subgroup findings from the glycemic-control meta-analysis and with RCTs in PCOS [37,38,48]. Overall, the preclinical basis for improved insulin signaling and glucose disposal is coherent, but clinical efficacy remains subgroup-dependent; mechanistic human studies should verify tissue exposure, target engagement, and durability.

### 4.4. Regulation of Lipid Metabolism

Quercetin regulates lipid metabolism by decreasing lipogenesis, increasing fatty-acid oxidation, modulating cholesterol transport, and reducing hepatic lipid deposition. In preclinical NAFLD models, quercetin attenuates steatosis through pathways involving VLDL assembly, lipophagy, AMPK/SIRT1 signaling, and gut–liver axis modulation [20,22,23]. In adipose tissue, quercetin can reduce inflammatory infiltration and promote thermogenic programming, supporting energy expenditure [21,30]. In clinical studies, quercetin’s lipid effects are modest but may be meaningful when integrated with improvements in inflammation, blood pressure, glycemic control, and liver fat [41,47]. Overall, effects on hepatic steatosis appear more consistent than effects on circulating lipids, suggesting that future studies should separate liver-specific outcomes from conventional lipid panels and relate both to quercetin exposure.

### 4.5. Enhancement of Endothelial Function and Vascular Effects

Endothelial dysfunction is a central bridge between MetS and cardiovascular disease. Quercetin improves vascular function by increasing nitric oxide bioavailability, reducing superoxide-mediated NO degradation, suppressing oxidized LDL-related injury, and modulating endothelial autophagy [26,33]. The activity of quercetin metabolites is especially relevant because circulating conjugates and microbial catabolites may reach higher systemic concentrations than the aglycone after oral intake [9,55]. The clinical antihypertensive signal observed in RCTs and meta-analyses suggests that vascular mechanisms are among the most translatable actions of quercetin [42,45]. Overall, vascular function and systolic blood pressure currently show the clearest clinical translation, although the contribution of individual conjugates and metabolites and the persistence of benefit after longer treatment remain unresolved.

### 4.6. Modulation of the Gut Microbiome

Quercetin and its glycosides undergo extensive interaction with the gut microbiota. Unabsorbed quercetin derivatives can be deglycosylated and degraded into smaller phenolic acids, while quercetin can alter microbial composition, increase SCFA-producing taxa, reduce endotoxin-producing bacteria, and strengthen barrier integrity [9,55]. In HFD-induced obese mice, quercetin improved gut-barrier function and shifted microbial communities and metabolites, linking microbiome remodeling with reduced inflammation and improved metabolic status [29]. These findings support a food-matrix perspective: quercetin’s effects may depend not only on systemic absorption but also on luminal exposure, microbial conversion, and cross-feeding interactions in the colon. Overall, microbiome modulation is a plausible mediator of quercetin action but remains predominantly preclinical; causal mediation, reproducibility across diets and populations, and the identity of active microbial products require targeted human studies.

### 4.7. Integrated Mechanistic Framework and Remaining Gaps

In a unified framework, quercetin and its host- and microbiota-derived metabolites may act on a coupled redox–inflammation–metabolism axis. Nrf2/HO-1 activation can lower the cellular ROS burden and thereby attenuate redox-sensitive NF-κB/NLRP3 activation; reduced inflammatory signaling can help preserve insulin-receptor/PI3K/Akt function, while AMPK/SIRT1 activation suppresses gluconeogenesis and lipogenesis, promotes fatty-acid oxidation and autophagy, and supports eNOS/NO-dependent vascular function. Concurrent improvement of gut-barrier integrity may lower LPS exposure, further attenuating NF-κB/NLRP3 signaling and metabolic inflammation. These links should be regarded as a proposed integrative model because most have been demonstrated separately in cell or animal systems rather than simultaneously at physiologically relevant human exposures. Causal pathway-interruption studies, tissue pharmacokinetics, and experiments using circulating conjugates and microbial metabolites are needed to determine which nodes are primary in humans [16,17,21,22,23,24,25,26,27,28,29,30,50,51].

## 5. Quercetin as a Food-Preservation Bioactive: Antimicrobial, Antioxidant, Photodynamic, and Active-Packaging Applications

Beyond its nutraceutical potential, quercetin is increasingly studied as a multifunctional food-preservation bioactive. Plant polyphenols are receiving growing attention as clean-label alternatives to synthetic preservatives because they can combine antioxidant, antimicrobial, and matrix-stabilizing functions [56]. The term food preservation is used here as an umbrella concept that includes direct antimicrobial and antioxidant effects in foods, inhibition of biofilms on processing surfaces, photodynamic decontamination, edible films/coatings, and active or intelligent packaging. Within this framework, active packaging is treated as a major technological strategy within the broader field of food preservation. Table 3 summarizes key studies, with emphasis on recent work from 2020 to 2026, whereas Table 4 provides a comparative appraisal of the advantages, limitations, and practical applicability of the main quercetin-based food-preservation strategies.

### 5.1. Antimicrobial and Antibiofilm Activity

Quercetin exhibits antimicrobial and antibiofilm effects against relevant foodborne pathogens, although efficacy is concentration-, matrix-, and strain-dependent. Recent studies demonstrate that quercetin suppresses Salmonella Typhimurium and Listeria monocytogenes biofilm formation on food-contact surfaces, including rubber, gloves, stainless steel, and silicone rubber [57,58]. These effects are associated with repression of virulence, quorum-sensing, motility, stress-response, and biofilm-related genes. The activity of quercetin-derived gut catabolites against Listeria further indicates that the antimicrobial relevance of quercetin may extend beyond the parent molecule to metabolites formed from quercetin-rich foods such as onions [62]. Because biofilms are major reservoirs for cross-contamination in food processing, quercetin’s antibiofilm activity may be particularly valuable as part of hurdle systems rather than as a stand-alone sanitizer.

### 5.2. Photodynamic and Hurdle-Preservation Strategies

Quercetin can act as a food-compatible photosensitizer under blue light, generating reactive oxygen species that damage bacterial membranes and enzymes. In buffer systems, quercetin-mediated antimicrobial photodynamic treatment reduced E. coli O157:H7 and L. monocytogenes through membrane-disrupting ROS production [59]. In apple juice, 405 nm blue light combined with quercetin achieved more than 5-log reductions of both pathogens while maintaining key quality attributes, especially at lower processing temperature [60]. Quercetin also shows promise in combined treatments, as illustrated by processed sausage studies where quercetin plus postbiotic metabolites improved biocidal and antibiofilm efficacy against Listeria and Salmonella [61]. These results position quercetin as a flexible component of nonthermal, clean-label preservation systems.

### 5.3. Antioxidant Protection and Stabilization of Food Quality

Quercetin is a strong chain-breaking antioxidant capable of delaying lipid oxidation, scavenging radicals, chelating metals, and protecting oxidation-sensitive food constituents. In real foods, however, antioxidant performance depends on partitioning, pH, water activity, protein binding, oxygen permeability, and interaction with other bioactives. Active films containing quercetin have reduced lipid oxidation markers such as TBARS and slowed quality deterioration in seafood, meat, and produce systems [64,66,70]. Because quercetin is relatively hydrophobic and light-sensitive, immobilization in biopolymers, nanocrystals, Pickering emulsions, or coated biodegradable films can improve controlled release and extend its antioxidant function during storage [63,66,73].

### 5.4. Edible Films, Coatings, and Active Packaging

The most active technological frontier for quercetin in food preservation is active packaging. Recent studies demonstrate incorporation of quercetin into gelatin, chitosan, kafirin, alginate, carrageenan, PLA, PBS/PVOH, polypropylene, and chitosan-PVA matrices [63,64,65,66,67,69,70,73,74,75]. These materials can provide antioxidant activity, UV shielding, antimicrobial action, pH-responsive spoilage indication, biodegradability, and controlled release. Particularly notable are 2025–2026 studies showing chitosan–quercetin films for fish-spoilage monitoring, quercetin nanocrystal/Pickering emulsion films for meat preservation, PLA + quercetin coatings on biodegradable films for avocado browning control, polypropylene membranes using quercetin and curcumin for shrimp freshness monitoring, and edible quercetin/orange-essential-oil films with antifungal activity against postharvest pathogens [69,70,73,74,75]. Collectively, these studies support active packaging as a core component of quercetin-based food-preservation strategies.

### 5.5. Food-Matrix and Sensory Considerations

Despite strong functionality, quercetin application in foods faces practical constraints. Its low water solubility can limit dispersion in aqueous foods, while its color, bitterness, astringency, and oxidation products may affect sensory quality. Matrix interactions can either enhance or reduce antimicrobial and antioxidant efficacy by controlling partitioning, release, and contact with microbial cells or oxidizable substrates. Therefore, future studies should move beyond model systems toward realistic food matrices, sensory validation, migration assessment, stability during processing, and regulatory compatibility. Bioavailability-focused formulation strategies developed for nutraceuticals—such as colloidal delivery, cyclodextrin inclusion, phytosomes, and self-emulsifying systems—may also inform food-preservation applications when edible, food-grade materials are used [11,13,76,77]. Another high-impact direction is sustainable recovery from agri-food by-products. Recent studies and reviews show that onion-skin waste is a quercetin-rich source, that polarity affects antioxidant performance in oil matrices, and that microwave/enzyme-assisted extraction can improve quercetin recovery from onion peel [78,79,80].

### 5.6. Comparative Evidence and Translational Maturity

Across the reviewed literature, quercetin-based preservation research is shifting from free-quercetin tests in model systems toward localized, controlled-release, and multifunctional films, coatings, and packaging prototypes evaluated in foods [57,58,59,60,61,62,63,64,65,66,67,68,69,70,71,72,73,74,75]. Direct antibiofilm use remains predominantly an in vitro surface-based concept [57,58,62]. Photodynamic treatment has progressed from buffer systems to apple juice, where reductions greater than 5 log were reported, but its performance in opaque or structurally heterogeneous foods remains uncertain [59,60]. Hurdle combinations are promising but are represented by limited food-matrix evidence [61]. Edible coatings and active/intelligent packaging have the broadest range of laboratory food applications, including meat, seafood, produce, and postharvest models [63,64,65,66,67,68,69,70,71,72,73,74,75]; nevertheless, most reports remain formulation-specific and laboratory-scale.

No strategy can yet be considered commercially validated on the basis of the reviewed evidence. Direct incorporation is operationally simple but constrained by solubility, sensory effects, and matrix binding. Photodynamic approaches offer rapid nonthermal inactivation but require adequate light delivery and process validation. Films and coatings enable localization and controlled release but introduce requirements related to mechanical performance, migration, stability, scalability, end-of-life management, and regulatory compliance. Hurdle systems may reduce the intensity required from each individual intervention, although interactions and reproducibility must be established for each food matrix. Consequently, comparative testing under standardized and commercially relevant storage and microbial-challenge conditions is now more important than generating additional proof-of-concept formulations alone [56,59,60,61,62,63,64,65,66,67,68,69,70,71,72,73,74,75,76,77].

## 6. Plant Metabolism and Production-Relevant Extraction and Microbial Synthesis

### 6.1. Biosynthesis and Metabolism in Plants

In plants, quercetin is produced through the phenylpropanoid–flavonoid pathway. Phenylalanine is converted through phenylalanine ammonia-lyase, cinnamate 4-hydroxylase, and 4-coumarate-CoA ligase to *p*-coumaroyl-CoA. Chalcone synthase and chalcone isomerase subsequently produce naringenin, which is converted by flavanone 3-hydroxylase to dihydrokaempferol. Flavonoid 3′-hydroxylase forms dihydroquercetin, and flavonol synthase catalyzes its final oxidation to quercetin [81,82]. Flavonol synthase isoforms can show tissue- and stimulus-dependent activity, contributing to spatial and environmental variation in quercetin accumulation [81].

Quercetin is subsequently subjected to position-specific glycosylation and other conjugation reactions that influence its accumulation and chemical form. Genetic evidence from *Arabidopsis thaliana* shows that impairment of the major flavonol 3-*O*-glycosyltransferases markedly decreases quercetin and total flavonol accumulation and alters feedback regulation of phenylpropanoid-pathway genes, indicating that glycosylation is an integral component of pathway regulation rather than merely a terminal modification [82]. Onion glucosyltransferases also exhibit position-specific activity toward quercetin [83]. Accordingly, onion tissues accumulate predominantly quercetin 4′-*O*-glucoside and quercetin 3,4′-*O*-diglucoside, whose relative abundance varies between bulbs and leaf blades, among cultivars, and throughout plant development [84]. These findings indicate that species, genotype, tissue, and developmental stage should be considered when selecting and characterizing raw materials for quercetin recovery.

### 6.2. Production-Relevant Extraction and Microbial Synthesis

From a production perspective, recovery from quercetin-rich plant by-products and heterologous microbial biosynthesis represent complementary routes. Onion-skin studies have evaluated process-intensification strategies such as microwave/enzyme-assisted extraction [80], pulsed-electric-field pretreatment followed by subcritical-water extraction [85], and water-based extraction followed by single-step liquid–liquid isolation [86]. Pulsed-electric-field pretreatment increased total quercetin recovery by 33.22% relative to the untreated extraction control, reaching 19.25 ± 0.77 mg/g dry onion skin under the optimized laboratory conditions [85]. In another study, liquid–liquid isolation yielded 10.88 ± 0.20 mg quercetin/g dry onion scales at 93 ± 2% purity and reduced solvent use per milligram of purified quercetin by approximately 600-fold relative to the column-chromatography comparator used in that study [86].

Microbial synthesis has also advanced through reconstruction and optimization of the plant flavonol pathway in engineered microorganisms. An engineered *Saccharomyces cerevisiae* platform produced quercetin directly from glucose, and optimization of precursor supply and pathway-limiting enzymes resulted in a reported titer of 930 mg/L in fed-batch fermentation [87]. These results demonstrate process feasibility and scale-up potential, but they do not by themselves establish commercial or industrial-scale performance. Comparative pilot-scale studies are still needed to evaluate feedstock variability, extraction or fermentation yield, glycoside-to-aglycone conversion, product purity, solvent and water recycling, batch consistency, operating cost, regulatory specifications, and life-cycle impacts before the relative industrial advantages of these routes can be established.

## 7. Conclusions and Future Directions

Quercetin is a mechanistically plausible multi-target bioactive for MetS, but support differs substantially by evidence level. Cellular and animal studies consistently indicate effects on redox, inflammatory, glucose- and lipid-metabolic, vascular, hepatic, and microbiota-related pathways. Human evidence is narrower: the most consistent signal is a modest reduction in systolic blood pressure, whereas findings for fasting glucose, lipids, inflammatory markers, endothelial function, and liver-fat outcomes are suggestive but heterogeneous, and evidence for weight loss and several other MetS endpoints is inconsistent [32,33,34,35,36,37,38,39,40,41,42,43,44,45,46,47,48,49]. Because the available trials are generally small and short and the tested formulations are not interchangeable, quercetin should currently be considered an investigational adjunct rather than a replacement for established MetS prevention or treatment.

For food preservation, the available evidence is predominantly experimental. In vitro studies and laboratory-scale food and packaging prototypes support antimicrobial, antibiofilm, photodynamic, antioxidant, controlled-release, and freshness-indication functions [57,58,59,60,61,62,63,64,65,66,67,68,69,70,71,72,73,74,75]. However, industrial-scale performance, long-term material stability, sensory acceptance, migration and toxicology, manufacturing cost, and regulatory compliance remain insufficiently evaluated. The current evidence therefore establishes technological feasibility but not commercial readiness.

Future work should address three linked levels. Experimental studies should identify active quercetin conjugates and microbial catabolites, test pathway causality at physiologically achievable exposures, and employ standardized food-challenge models. Clinical studies should use adequately powered, preregistered, longer-duration trials with well-characterized formulations, pharmacokinetic verification, standardized MetS endpoints, phenotype stratification, and prospective safety monitoring. Translational food-technology and production studies should validate pilot-scale manufacture, extraction or fermentation yield, product purity, controlled release, migration, sensory performance, shelf-life efficacy, toxicology, resource use, environmental performance, cost, and regulatory acceptability under commercial storage and distribution conditions. Sustainable recovery from quercetin-rich by-products and microbial synthesis should be compared within these efficacy, safety, and scale-up requirements rather than as isolated production objectives.

## Figures and Tables

**Table 1:** In vivo studies evaluating the effect of quercetin on the prevention and treatment of metabolic syndrome and associated disorders.

Disorder	Animal Model (Total *n*)	Study Details	Experimental Findings	Reference
Metabolic syndrome	Male Wistar rats (*n* ≈ 48)	High-carbohydrate/high-fat diet for 16 weeks; quercetin administered during the intervention phase.	Quercetin reduced visceral adiposity, systolic blood pressure, plasma triglycerides, and hepatic inflammation; improved cardiac and hepatic structure, supporting multi-target effects in diet-induced MetS.	[18]
Obesity/inflammation	Obese Zucker rats (*n* = 24)	Genetic obesity model; chronic quercetin supplementation.	Quercetin improved dyslipidemia and insulin resistance-related markers, lowered inflammatory mediators, and attenuated obesity-associated oxidative stress.	[19]
Hepatic steatosis	C57BL/6J mice (*n* ≈ 32)	Western-style diet with chronic dietary quercetin.	Dietary quercetin attenuated hepatic fat accumulation and modulated lipid-metabolism genes, supporting prevention of diet-induced hepatic steatosis.	[20]
Obesity	C57BL/6J mice (*n* ≈ 40)	High-fat diet with quercetin supplementation.	Quercetin reduced adipose-tissue macrophage infiltration and inflammatory tone, with mechanisms involving AMPKα1/SIRT1 signaling.	[21]
Hepatic steatosis/gut-liver axis	C57BL/6J mice (*n* ≈ 36)	High-fat diet-induced hepatic steatosis; quercetin administered as a dietary intervention.	Quercetin improved hepatic steatosis and inflammation by correcting gut dysbiosis and reducing gut–liver axis activation.	[22]
Hepatic steatosis	C57BL/6J mice (*n* ≈ 40)	High-fat diet-induced hepatic steatosis; quercetin intervention.	Quercetin promoted hepatic VLDL assembly and lipophagy through the IRE1α/XBP1s pathway, reducing hepatic lipid accumulation.	[23]
Diabetes	Fructose/STZ-induced diabetic rats (*n* ≈ 36)	Quercetin at 25 or 50 mg/kg for 28 days.	Quercetin lowered fasting glucose and HbA1c, improved carbohydrate-metabolism enzymes, and increased pancreatic antioxidant defenses.	[24]
Hypertension	Spontaneously hypertensive rats (*n* ≈ 48)	Phenolic metabolites of quercetin administered at low doses.	A metabolite mixture decreased elevated blood pressure, indicating that circulating quercetin metabolites may contribute to vascular effects.	[25]
Hypertension/endothelial function	Spontaneously hypertensive rats (*n* = 40)	Quercetin 10 mg/kg/day by gavage for 6 weeks.	Quercetin lowered blood pressure and improved endothelial function, linked to enhanced vascular autophagy and NO-related signaling.	[26]
Type 2 diabetes complications	Obese diabetic Sprague-Dawley rats (*n* ≈ 40)	High-fat diet/STZ model; quercetin 300 mg/kg in comparison with crocin.	Quercetin improved glucose and lipid alterations and reduced renal and hepatic injury, partly through AMPK/autophagy-associated mechanisms.	[27]
Atherosclerosis	ApoE−/− mice (*n* ≈ 40)	High-fat diet with quercetin 100 mg/kg body weight for 16 weeks.	Quercetin reduced atherosclerotic plaque burden and inflammatory signaling by suppressing the Galectin-3/NLRP3 pathway.	[28]
Obesity/gut microbiota	C57BL/6J mice (*n* ≈ 48)	High-fat diet; quercetin 50 mg/kg body weight for 20 weeks.	Quercetin decreased weight gain and adiposity, improved glucose tolerance, strengthened the gut barrier, and shifted microbiota and metabolites toward a healthier profile.	[29]
Obesity/thermogenesis	HFD-induced obese mice (*n* ≈ 36)	High-fat diet with 1% quercetin supplementation.	Quercetin reduced body weight and cholesterol and stimulated non-shivering thermogenesis through activation of brown adipose tissue and browning of white adipose tissue.	[30]
Hypertension	L-NAME-induced hypertensive rats (*n* ≈ 36)	Acute intravenous quercetin administration in nitric-oxide synthase inhibition-induced hypertension.	Acute quercetin lowered arterial blood pressure and improved vascular responses, supporting a rapid vasomodulatory component.	[31]

Abbreviations: AMPK = AMP-activated protein kinase; ApoE−/− = apolipoprotein E knockout; HbA1c = glycated hemoglobin; HFD = high-fat diet; IRE1α = inositol-requiring enzyme 1 alpha; L-NAME = Nω-nitro-L-arginine methyl ester; MetS = metabolic syndrome; NLRP3 = NOD-like receptor family pyrin domain containing 3; NO = nitric oxide; STZ = streptozotocin; VLDL = very-low-density lipoprotein; XBP1s = spliced X-box binding protein 1.

**Table 2:** Clinical trials and meta-analyses evaluating the effect of quercetin or quercetin-rich formulations on metabolic syndrome and associated disorders.

Disorder	Quercetin Source	Participants (Total *n*)	Study Details	Experimental Findings	Reference
Hypertension	Pure quercetin	Adults with stage 1 hypertension or prehypertension (*n* = 41)	Randomized, double-blind, placebo-controlled crossover trial; quercetin 730 mg/day for 28 days.	Quercetin reduced systolic and diastolic blood pressure in hypertensive participants, providing early clinical support for vascular effects.	[32]
Dyslipidemia/blood pressure	Pure quercetin	Overweight/obese adults with high-CVD-risk phenotype (*n* = 93)	Double-blind trial; quercetin 150 mg/day for 6 weeks.	Quercetin reduced systolic blood pressure and oxidized LDL in higher-risk participants; lipid effects were modest and phenotype-dependent.	[33]
Type 2 diabetes/inflammation	Pure quercetin	Women with type 2 diabetes (*n* ≈ 70)	Double-blind RCT; quercetin supplementation for 10 weeks.	Reported improvements in selected cardiovascular and inflammatory biomarkers, supporting quercetin as an adjunct dietary bioactive.	[34]
Prehypertension/endothelial function	Onion-skin extract rich in quercetin	Overweight-to-obese adults with prehypertension (*n* = 70)	Randomized double-blind crossover trial; quercetin-rich onion-skin extract.	The extract lowered 24 h ambulatory blood pressure and improved vascular endpoints in a high-risk phenotype.	[35]
Cardiometabolic health	Pure quercetin/epicatechin	Healthy adults (*n* = 37)	Randomized double-blind crossover trial comparing isolated flavonoids.	Quercetin had limited effects on most endpoints but contributed evidence on translation from purified flavonoids to vascular and metabolic outcomes.	[36]
Insulin resistance/PCOS	Pure quercetin	Women with PCOS (*n* = 84)	Randomized double-blind placebo-controlled trial; quercetin 1 g/day for 12 weeks.	Quercetin improved adiponectin-mediated insulin sensitivity and endocrine-metabolic markers in PCOS.	[37]
PCOS/obesity	Pure quercetin	Overweight or obese women with PCOS (*n* = 72)	Randomized trial; quercetin supplementation for 12 weeks.	Quercetin improved metabolic and hormonal parameters and modulated resistin-related pathways.	[38]
Endothelial function	Enzymatically modified isoquercitrin	Volunteers at risk of CVD (*n* = 70)	Randomized controlled human study using a more bioavailable quercetin glycoside.	Isoquercitrin improved endothelial function, suggesting that bioavailability-enhanced forms may yield stronger vascular responses.	[39]
Metabolic syndrome	Pure quercetin	Elderly patients with MetS (*n* ≈ 60)	Randomized placebo-controlled trial; 240 mg/day quercetin for 3 months.	Quercetin decreased body weight, BMI, systolic and diastolic blood pressure, total cholesterol, LDL-C, fasting insulin, and 2 h OGTT glucose.	[40]
Lipids/inflammation	Pure quercetin	RCTs in MetS-related disorders	Systematic review and meta-analysis of randomized controlled trials.	Quercetin supplementation decreased total cholesterol, LDL-C and CRP, while effects on triglycerides, HDL-C and several cytokines were inconsistent.	[41]
Blood pressure	Quercetin supplements	Adults across RCTs	Meta-analysis focused on blood-pressure outcomes.	Quercetin significantly reduced systolic blood pressure, with smaller or less consistent effects on diastolic blood pressure depending on subgroup.	[42]
Oxidative stress	Quercetin supplements	RCTs in adults	Systematic review and meta-analysis of oxidative stress markers.	Quercetin improved selected antioxidant-capacity indices, although effects on MDA and TAC were not uniformly significant.	[43]
Cardiometabolic factors	Quercetin supplements	Meta-analyses of RCTs	Umbrella review of meta-analyses.	The most consistent evidence supported reductions in systolic blood pressure and insulin, whereas evidence for lipids, inflammation and anthropometry was more uncertain.	[44]
Metabolic syndrome components	Quercetin supplements	20 RCTs, *n* = 1164	Systematic review and dose–response meta-analysis of RCTs.	Quercetin reduced fasting blood glucose and systolic blood pressure but showed no pooled effect on triglycerides, HDL-C, waist circumference or diastolic blood pressure.	[45]
NAFLD (original trial terminology)	Pure quercetin	Participants diagnosed with NAFLD under the original trial criteria (*n* = 41 randomized; 36 completed)	Randomized, double-blind, placebo-controlled crossover trial; 500 mg/day quercetin for 12 weeks.	Quercetin reduced intrahepatic lipid content measured by MRI-PDFF and mildly reduced body weight and BMI, with no intervention-related adverse events.	[46]
MASLD	Quercetin supplements	7 RCTs, *n* = 540	Systematic review and meta-analysis through January 2025.	Quercetin improved liver enzymes, CRP and lipid profile in MASLD, but certainty remained low to moderate and no consistent effect was observed for BMI or TNF-α.	[47]
Glycemic control	Quercetin supplements	RCTs in MetS-related disorders	Systematic review and meta-analysis.	Overall effects on fasting glucose, HOMA-IR and HbA1c were limited, but subgroup analyses suggested benefits at ≥500 mg/day or ≥8 weeks.	[48]
Body weight	Quercetin supplements	9 RCTs, *n* = 525	Pooled analysis of RCTs examining body-weight management.	No robust overall weight-loss effect, although subgroup analyses suggested modest benefit with longer duration or higher doses.	[49]

Abbreviations: BMI = body mass index; CRP = C-reactive protein; CVD = cardiovascular disease; HbA1c = glycated hemoglobin; HDL-C = high-density lipoprotein cholesterol; HOMA-IR = homeostatic model assessment of insulin resistance; LDL-C = low-density lipoprotein cholesterol; MASLD = metabolic dysfunction-associated steatotic liver disease; MDA = malondialdehyde; MRI-PDFF = magnetic resonance imaging proton-density fat fraction; NAFLD = nonalcoholic fatty liver disease (term retained for the original trial); OGTT = oral glucose tolerance test; PCOS = polycystic ovary syndrome; RCT = randomized controlled trial; TAC = total antioxidant capacity; TNF-α = tumor necrosis factor alpha.

**Table 3:** Key studies on quercetin as a food-preservation bioactive, highlighting antimicrobial, antibiofilm, antioxidant, photodynamic, edible-film/coating, and active-packaging effects.

Study Type	Food Matrix or Model System	Quercetin Form or Delivery Method	Main Observed Effect	Reference
In vitro/food-contact surfaces	Rubber and glove food-contact surfaces inoculated with *Salmonella* Typhimurium	Free quercetin as antibiofilm agent	Quercetin inhibited biofilm formation and downregulated genes associated with virulence, stress response, and quorum sensing, supporting its use to reduce cross-contamination risk.	[57]
In vitro/food-contact surfaces	Stainless steel, silicone rubber and glove coupons inoculated with *Listeria monocytogenes* mixed cultures	Free quercetin	Quercetin reduced *Listeria* biofilm formation and repressed virulence-associated traits across relevant processing surfaces.	[58]
Nonthermal antimicrobial processing	Phosphate-buffered saline model systems inoculated with *E. coli* O157:H7 and *L. monocytogenes*	Quercetin as photosensitizer under 405 nm blue light	Quercetin-mediated antimicrobial photodynamic treatment produced large log reductions by generating ROS that damaged bacterial membranes.	[59]
Food application/beverage	Apple juice inoculated with *E. coli* O157:H7 and *L. monocytogenes*	Quercetin-mediated photodynamic inactivation with 405 nm blue light	Treatment achieved >5-log reductions while maintaining juice quality, especially when conducted at lower temperature.	[60]
Food application/meat	Processed meat sausage inoculated with *Listeria monocytogenes* and *Salmonella* Typhimurium	Quercetin alone and combined with Lactobacillus curvatus B67-derived postbiotic	Combination treatments enhanced biocidal and antibiofilm activity, indicating a hurdle approach for processed meat safety.	[61]
In vitro/metabolite mechanism	Listeria monocytogenes exposed to gut catabolites of quercetin 4′-glucoside	3-hydroxyphenylacetic acid and 3,4-dihydroxyphenylacetic acid	Quercetin-derived catabolites damaged membranes and suppressed biofilm formation, extending preservation relevance beyond the parent compound.	[62]
Active packaging film	Gelatin films containing quercetin, lactoferrin and chitosan nanofibers	Immobilized quercetin in gelatin-based nanocomposite film	Films combined antioxidant, antimicrobial, ammonia-sensitive color response and biodegradability, demonstrating multifunctional active and intelligent packaging.	[63]
Active packaging film/seafood	Cod during cold storage	Kafirin-quercetin film	Quercetin improved film functionality and restrained microbial growth, TVB-N accumulation and lipid oxidation during refrigerated cod storage.	[64]
Active packaging film	Gelatin/chitosan films	Genipin-crosslinked matrix with rosemary essential oil and quercetin	Quercetin contributed UV-barrier, antioxidant and antibacterial properties in a biodegradable active film.	[65]
Edible coating/meat	Pork meat stored under refrigeration	Alginate coating charged with hydroxyapatite complexes containing lactoferrin and quercetin	The coating slowed microbial growth and TVB-N formation, extending pork shelf life during 15 days at 4 °C.	[66]
Active packaging membrane	Polypropylene membranes for food packaging	Curcumin- and quercetin-functionalized PP membranes	Bioactive membranes showed antibacterial behavior and pH-responsive color changes, supporting active/smart packaging applications.	[67]
Active packaging film/produce	Baby carrots during refrigerated storage	Nano-reinforced chitosan films with quercetin and cellulose nanocrystals	Quercetin-containing films improved antioxidant/antimicrobial properties and helped maintain baby-carrot quality over storage.	[68]
Smart active packaging/fish	Crucian carp spoilage monitoring	Chitosan-quercetin active film	Quercetin enhanced antibacterial and antioxidant activity, enabled pH-responsive spoilage detection and biodegraded within approximately 70 days.	[69]
Controlled-release active packaging/meat	Fresh meat preservation	Chitosan-PVA films with quercetin nanocrystals and cinnamon essential oil Pickering emulsions	The system improved controlled release, mechanical performance, antimicrobial activity, UV shielding and meat shelf-life extension.	[70]
Active packaging/meat	Fresh meat preservation	Chitosan/PVA film reinforced with quercetin self-assembled nanocrystals	Quercetin nanocrystals reinforced film structure and supported antioxidant and preservation performance in fresh meat.	[71]
Edible film/meat	Goat meat burger patties	κ-carrageenan edible films enriched with quercetin	Quercetin-containing films supported refrigerated shelf-life extension through quality preservation, although antimicrobial activity depended on matrix and storage conditions.	[72]
Biodegradable coating/produce	Avocado browning model and food simulants	PLA + quercetin coatings on PBS/PVOH films	Sealable biodegradable films released quercetin within migration limits, retained antioxidant activity and delayed browning, supporting food-preservation packaging.	[73]
Smart packaging/seafood freshness	Fresh shrimp and ammonia spoilage simulants	Polypropylene membrane containing curcumin and quercetin	The membrane combined barrier properties, bioactive release and progressive color change to spoilage-related volatile compounds.	[74]
Edible antifungal film/postharvest	Colletotrichum gloeosporioides model for fruit and vegetable contamination	Edible polysaccharide film with quercetin and orange essential oil	The best formulation inhibited fungal growth and maintained favorable mechanical/stability properties, indicating potential for postharvest protection.	[75]

Abbreviations: PBS/PVOH = poly(butylene succinate)/poly(vinyl alcohol); PLA = poly(lactic acid); PP = polypropylene; PVA and PVOH = poly(vinyl alcohol); ROS = reactive oxygen species; TVB-N = total volatile basic nitrogen; UV = ultraviolet.

**Table 4:** Comparative appraisal of quercetin-based food-preservation strategies.

Strategy	Evidence in Reviewed Literature	Main Advantages	Principal Limitations	Relative Maturity and Next Step
Direct antimicrobial and antibiofilm	Food-contact coupons and in vitro pathogen models [57,58,62]	Simple formulation; useful mechanistic evidence; potential adjunct for surface control	Concentration-, strain-, and matrix-dependent; limited residual-efficacy and real-food evidence	Early proof of concept; validate in processing environments and food matrices
Photodynamic treatment	Buffer models and one apple-juice application with large microbial reductions [59,60]	Rapid, nonthermal inactivation	Requires adequate light access; depends on optical properties, temperature, and photosensitizer stability	Early food-matrix validation; test opaque/solid foods and pilot-scale equipment
Hurdle combinations	Processed-sausage model combining quercetin and a postbiotic [61]	Potentially combines mechanisms and reduces intensity of individual treatments	Interactions, formulation, and reproducibility are system-specific; small evidence base	Exploratory; replicate across pathogens and food matrices
Edible films and coatings	Meat, seafood, produce, and postharvest storage models [64,66,68,72,73,75]	Localized delivery; antioxidant/antimicrobial functionality; potential biodegradability	Sensory effects, mechanics, release/migration, moisture sensitivity, and scale-up	Laboratory food validation; pilot manufacture plus shelf-life, sensory, and migration testing
Active and intelligent packaging	Multifunctional prototypes with controlled release or freshness indication [63,65,67,69,70,71,74]	Controlled release, UV protection, spoilage indication, and multifunctionality	Indicator calibration/stability, migration, manufacturing consistency, cost, regulation, and end of life	Laboratory-prototype stage; validate sensor accuracy, safety, scalability, and compliance

Note: Relative maturity represents a qualitative narrative appraisal based on the directness and realism of the cited evidence; it is not a formal technology-readiness assessment.

## Data Availability

No new data were created or analyzed in this study. Data sharing is not applicable to this article.
